# Long-term efficacy of neuromodulation in chronic pain management: a single-center retrospective study

**DOI:** 10.3389/fpain.2026.1737302

**Published:** 2026-02-02

**Authors:** Filip Blazek, David Krahulik, Lumir Hrabalek, Martin Gabrys

**Affiliations:** Department of Neurosurgery, University Hospital and Faculty of Medicine and Dentistry, Olomouc, Czechia

**Keywords:** chronic pain, neuromodulation, occipital stimulation, pain management, spinal cord stimulation

## Abstract

**Objectives:**

Neuromodulation is an advanced therapy for managing chronic pain by modulating nerve activity through electrical stimulation. This study evaluates the clinical outcomes of neuromodulation therapies, including spinal cord stimulation (SCS), occipital nerve stimulation (ONS), and peripheral nerve field stimulation (PNFS) in patients with refractory chronic pain.

**Methods:**

A retrospective analysis was conducted at the Department of Neurosurgery, Department of Neurosurgery, University Hospital Olomouc, on 70 patients who underwent neuromodulation therapy between 2019 and 2024. Indications included Persistent Spinal Pain Syndrome Type 2 (PSPS-T2), Persistent Spinal Pain Syndrome Type 1 (PSPS-T1), Complex Regional Pain Syndrome (CRPS), Failed Neck Surgery Syndrome, and neuropathic pain. Pain relief was assessed using the Visual Analog Scale (VAS) before and after implantation. Only patients with a successful trial stimulation who proceeded to permanent implantation were included in the analysis, and outcomes aree therefore interpreted as descriptive results in a responder-selected cohort.

**Results:**

Patients experienced significant pain reduction post-implantation. Among PSPS-T2 patients (*n* = 40), mean VAS scores decreased from 5.58 to 1.80 (67.2% reduction). Similar trends were observed in PSPS-T1 (64.5% reduction), CRPS (85% reduction), and other conditions, with an overall mean pain reduction of 58.3%. Percutaneous electrodes and non-rechargeable implantable pulse generators (IPGs) were most commonly used.

**Conclusion:**

In this single-center, retrospective real-world cohort, neuromodulation was associated with clinically meaningful pain reduction among patients who responded to trial stimulation. These data primarily illustrate contemporary neuromodulation practice and outcomes in a Central European academic center.

## Introduction

Neuromodulation is an advanced therapeutic technique that involves the targeted delivery of electrical or chemical stimuli to modulate nervous system activity, offering relief for a variety of neurological, pain-related, and functional disorders (1). By influencing nerve signaling, neuromodulation can either enhance or inhibit neural activity, thereby addressing conditions that are often resistant to conventional medical or surgical treatments (2). Over the years, this field has expanded significantly, with various techniques being developed to target specific neural structures, improving both efficacy and patient outcomes. Among the most widely used neuromodulation techniques are spinal cord stimulation (SCS), occipital nerve stimulation (ONS), peripheral nerve field stimulation (PNFS), and sacral neuromodulation (SNM), each of which has distinct mechanisms and clinical applications (3).

Spinal cord stimulation (SCS) involves the implantation of electrodes along the spinal cord to deliver controlled electrical impulses, which interfere with pain signaling pathways. This technique is primarily used for the management of chronic pain conditions, such as failed back surgery syndrome, complex regional pain syndrome, and neuropathic pain. By modifying pain perception at the spinal level, SCS provides significant pain relief and can reduce the reliance on opioid medications, thereby improving patients' quality of life (4, 5).

Occipital nerve stimulation (ONS) is a neuromodulation technique that targets the occipital nerves, which are implicated in certain chronic headache disorders. This method has shown promise in treating refractory chronic migraines, cluster headaches, and occipital neuralgia. By modulating the sensory pathways involved in headache pathophysiology, ONS can provide relief for patients who have not responded to standard pharmacological treatments (6).

Peripheral nerve field stimulation (PNFS) is a minimally invasive technique that involves placing electrodes near peripheral nerves or in the subcutaneous tissue overlying the painful area. It is particularly effective for treating localized neuropathic pain conditions, such as post-surgical pain, post-herpetic neuralgia, and other forms of focal chronic pain. PNFS offers a targeted approach to pain management by disrupting abnormal pain signaling in the peripheral nervous system (7).

Sacral neuromodulation (SNM) is used primarily for treating functional disorders of the pelvic organs, including overactive bladder, urinary retention, fecal incontinence, and certain forms of pelvic pain. By delivering mild electrical stimulation to the sacral nerves, SNM helps restore normal communication between the brain and the pelvic organs, improving symptoms in patients with refractory urological and gastrointestinal dysfunction (8).

Together, these neuromodulation techniques offer a promising alternative for individuals suffering from chronic pain and functional disorders that do not respond to conventional therapies. As research advances, further refinement of these methods, along with the development of new technologies, continues to enhance their effectiveness and expand their clinical applications (9).

This study presents a comprehensive evaluation of our clinical experience, detailing patient selection criteria, implantation techniques, and therapeutic outcomes. By contextualizing our findings within the broader field of neuromodulation, we aim to inform of best practices and contribute to ongoing advancements in pain management.

## Methods

This study was conducted at the Department of Neurosurgery at University Hospital Olomouc, Czech Republic to evaluate the effectiveness of neuromodulation therapy in managing chronic pain conditions. A retrospective analysis was performed on patients who underwent neuromodulation procedures between 2019 and 2024. Data was collected from institutional medical records, ensuring patient anonymity and compliance with ethical guidelines.

Patients included in this study received various neuromodulation therapies, including spinal cord stimulation (SCS), peripheral nerve field stimulation (PNFS), occipital nerve stimulation (ONS. The primary indication for therapy was Persistent Spinal Pain Syndrome Type 2 (PSPS-T2), formerly known as Failed Back Surgery Syndrome (FBSS), which refers to chronic pain that persists despite spinal surgery. Other indications included but were not limited to Persistent Spinal Pain Syndrome Type 1 (PSPS-T1), Failed Neck Surgery Syndrome, Complex Regional Pain Syndrome (CRPS), chronic neuropathic pain following spinal adhesive arachnitis after Lyme disease, migraine, fecal incontinence, thoracic pain following xyphoid exstirpation, post-viral myelitis, refractory angina pectoris, and neuralgias. The selection criteria for neuromodulation implantation were based on established clinical guidelines and multidisciplinary team recommendations.

Electrode placement was performed using either percutaneous or paddle electrodes, and patients were implanted with either rechargeable or non-rechargeable implantable pulse generators (IPG). A trial stimulation phase was conducted prior to permanent implantation to assess therapeutic efficacy. Pain intensity was assessed using the Visual Analog Scale (VAS) prior to trial stimulation and at follow-up visits after permanent implantation. For the purposes of this analysis, the post-implantation VAS value used corresponded to the most recent documented outpatient follow-up at a minimum of 6 months after implantation. Median follow-up duration was 14 months (interquartile range 9–26 months).

Trial stimulation was performed in all candidates prior to permanent implantation. Trial duration typically ranged from 5 to 14 days depending on indication and patient tolerance. A successful trial was defined as a minimum subjective pain reduction of approximately 50% and/or meaningful functional improvement as reported by the patient during multidisciplinary evaluation. Only patients meeting these criteria proceeded to permanent implantation.

Data analysis was primarily descriptive due to the retrospective design, heterogeneous indications, and small subgroup sizes. Pre- and post-implantation VAS scores were compared using paired tests where appropriate after visual inspection of distributions. Given the exploratory nature of subgroup analyses, no adjustment for confounders or multiple comparisons was performed, and results are presented to illustrate observed trends rather than infer causality. For transparency, measures of central tendency are reported without confidence intervals or formal hypothesis testing, as the study was not designed to support inferential statistical conclusions.

Patients without documented VAS scores at or beyond 6 months after implantation were excluded from outcome analysis. Loss to follow-up was limited to routine outpatient attrition and no formal imputation of missing data was performed.

## Results

A total of 70 patients were included in the study after excluding those with unsuccessful trial phases, with a mean age of 57.7 years (range: 36–79). The majority of patients received spinal cord stimulation (SCS) for Persistent Spinal Pain Syndrome Type 2 (PSPS-T2), a condition characterized by chronic pain persisting after spinal surgery.

Subgroup analyses were exploratory in nature, as several diagnostic categories contained a limited number of patients and were not powered for formal statistical comparison.

Among the PSPS-T2 group (*n* = 40), the mean pre-implantation VAS score was 5.58, which decreased to 1.80 post-implantation, reflecting a mean pain reduction of 67.2%. Similarly, patients with PSPS-T1 (*n* = 11) experienced a reduction from a mean pre-implantation VAS of 5.64–1.92, yielding an average pain reduction of 64.5%.

Patients with Complex Regional Pain Syndrome (CRPS) (*n* = 3) had an initial VAS score of 6.67, which decreased to 1.00 post-implantation, corresponding to a pain reduction of 85%.

Those diagnosed with Failed Neck Surgery Syndrome (*n* = 3) showed a pre-implantation VAS of 5.00 and a post-implantation VAS of 2.13, resulting in a 56.7% reduction in pain intensity. Meanwhile, patients with chronic neuropathic pain following spinal adhesive arachnitis after Lyme disease (*n* = 2) experienced a 60% reduction in pain, with VAS scores dropping from 5.5 to 2.25.

Patient with unspecific peripheral pain who underwent PNFS (*n* = 3) showed a decrease from a VAS of 4–1.5, also resulting in a significant reduction.

The biggest decrease of VAS was seen in patients with ONS implanted (*n* = 4) for migraine and occipital neuralgia, with an overall increase of over 84% from average VAS of 8.43–1.3.

The most commonly used electrode type was percutaneous, with most of the patient having 2 electrodes implanted. Implantable pulse generators (IPG) included both rechargeable and non-rechargeable systems, with non-rechargeable IPGs being the most frequently used.

Procedure-related complications were infrequent. One patient developed a superficial surgical site infection requiring antibiotic therapy without device explantation. Two patients required surgical revision for lead migration. No cases of permanent neurological deficit or device-related mortality were observed during follow-up.

**Figure 1:**
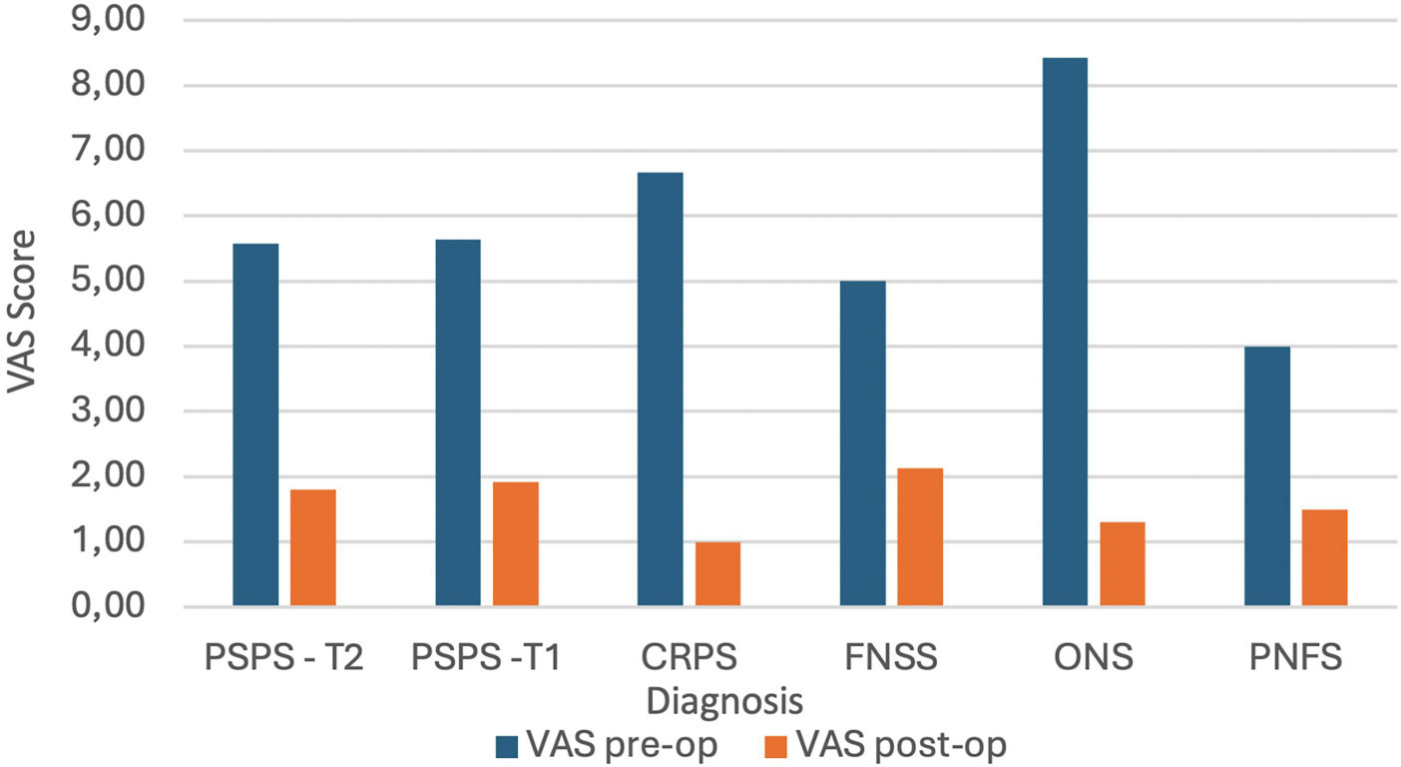
Table showing overall copmarison of VAS pre- and post- implant.

Pain relief, as measured by the Visual Analog Scale (VAS), showed a significant reduction following implantation across all groups (Figure 1). The average percentage reduction in pain intensity across all patients was 62.3%, supporting the effectiveness of neuromodulation therapy for a variety of chronic pain conditions.

These findings suggest that neuromodulation therapy, particularly SCS, is associated with clinically meaningful pain reduction among patients who successfully undergo trial stimulation.

## Discussion

The results of this study confirm that neuromodulation therapy, particularly spinal cord stimulation, is a viable treatment option for patients suffering from chronic pain conditions (10). The significant reduction in pain intensity observed in our cohort aligns with previous studies (11–13) that have demonstrated the efficacy of neuromodulation in patients with Persistent Spinal Pain Syndrome Type 2 and other chronic pain syndromes. The effectiveness of this therapy is further reinforced by the substantial decrease in Visual Analog Scale scores post-implantation. Importantly, this cohort includes only patients who demonstrated a successful response during trial stimulation, which inherently inflates observed effect sizes and limits generalizability to the broader population of neuromodulation candidates. Additional limitations include heterogeneity of indications, stimulation modalities, and programming strategies, as well as reliance on VAS as the primary outcome measure without standardized functional or quality-of-life instruments.

Despite the promising outcomes, a notable percentage of patients (10.3%) experienced an unsuccessful trial phase, indicating that patient selection and optimization of stimulation parameters remain crucial to achieving positive outcomes (14). Factors such as electrode placement, stimulation settings, and underlying pathology may influence the variability in patient responses to neuromodulation therapy (15). Further research is necessary to refine selection criteria and optimize treatment protocols to improve overall success rates. As pain outcomes were derived from routine clinical follow-up rather than blinded assessment, placebo effects, reporting bias, and patient–physician interaction effects cannot be excluded, particularly in earlier follow-up periods.

Additionally, while the majority of patients in our study benefited from pain relief, the long-term sustainability of these outcomes remains an important consideration. Future studies should incorporate extended follow-up periods to assess long-term efficacy, durability of pain relief, and potential complications associated with implantable devices. Moreover, cost-effectiveness analyses should be conducted to determine the economic feasibility of neuromodulation therapy in routine clinical practice.

Despite these limitations, this study provides a transparent real-world overview of neuromodulation practice within a Central European academic center, a region that remains underrepresented in the neuromodulation literature. The observed outcomes are broadly consistent with results reported in prospective trials and may serve as a benchmark for comparable healthcare systems.

## Conclusion

This study supports the role of neuromodulation therapy as an effective intervention for chronic pain conditions, particularly in patients with PSPS-T2 and other refractory pain syndromes. The significant reduction in pain intensity following implantation highlights the therapeutic potential of spinal cord stimulation and related neuromodulation techniques. However, careful patient selection, optimized device programming, and long-term monitoring are essential to maximizing treatment benefits. Future research should focus on improving patient selection criteria, refining stimulation parameters, and evaluating long-term outcomes to further enhance the efficacy and applicability of neuromodulation therapy.

## Data Availability

The raw data supporting the conclusions of this article will be made available by the authors, without undue reservation.
